# Amyloidosis presenting as bilateral transudative pleural effusions with normal cardiac investigations: a case report

**DOI:** 10.4076/1757-1626-2-6963

**Published:** 2009-07-21

**Authors:** James H Briggs, William G Singleton, Margaret M Burke, Lorraine A Hart, Robert J Parker

**Affiliations:** 1 Department of Respiratory Medicine, Heatherwood and Wexham Park Hospitals NHS Foundation Trust, King Edward VII HospitalWindsor, Berkshire, SL4 3DPUK; 2 Department of Histopathology, Royal Brompton and Harefield NHS TrustHarefield, Middlesex, UB9 6JHUK

## Abstract

A 66-year-old man with a diagnosis of monoclonal gammopathy of unknown significance was referred for investigation of bilateral transudative pleural effusions by the cardiology team. Echocardiography, myocardial perfusion scanning and left heart catheterisation were all normal or non diagnostic. Given significant occupational asbestos exposure in his twenties he underwent thoracoscopic pleural biopsy. This showed fibrous inflammation only. He subsequently developed proteinuria and peripheral oedema. Reanalysis of the pleural biopsy specimen for amyloidosis was positive. Pleural disease is an uncommon presentation of systemic amyloidosis. The aetiology of the pleural effusions is unclear and is not simply a consequence of cardiac or renal impairment.

## Case presentation

**Figure 1.**
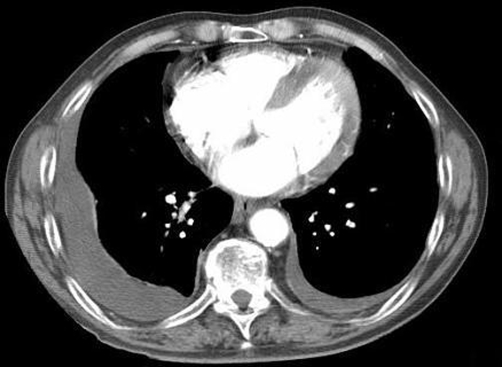
Computed tomography of the chest showing bilateral pleural effusions without other significant thoracic disease.

**Figure 2.**
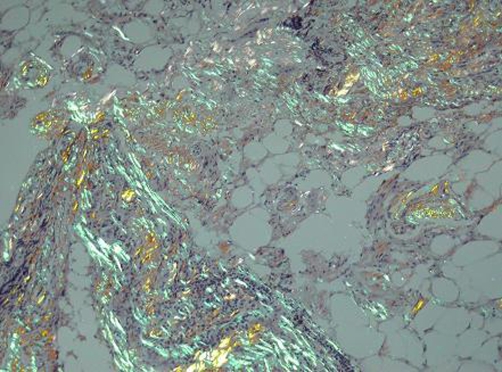
Congo red stain of pleural biopsy specimen showing the apple green birefringence of amyloid when seen under polarised light.

A 66-year-old white British man was referred from the cardiology team to the respiratory out-patient clinic for investigation of progressive breathlessness, unexplained bilateral pleural effusions and weight loss. Echocardiography had shown good left and right ventricular function, no significant valvular disease, normal E/A ratio and normal cardiac structure. No reversible ischaemia was seen at myocardial perfusion scanning. Left heart catheterisation showed only minor (30%) stenosis in the left anterior descending coronary artery and normal left ventriculogram. Medical history revealed hypertension, IgG kappa monoclonal gammopathy of undetermined significance (MGUS) for 5 years and strong occupational asbestos exposure whilst working in the building trade in his twenties. Pleural fluid analysis on two occasions demonstrated a transudative effusion by Light’s criteria (pleural protein 30 g/l, serum protein 91 g/l and lactate dehydrogenase 61 IU/l). There were no malignant cells on cytological examination. Thyroid function tests were within the normal reference ranges. Computed tomography of the chest showed only bilateral pleural effusions with no evidence of bronchial malignancy, prior asbestos exposure, interstitial disease, pulmonary embolus or pleural malignancy (Figure 1). He underwent video assisted thoracoscopic pleural examination and talc pleurodesis. The operative note reported ‘abnormal nodular pleural change’ which was biopsied. This showed non-specific chronic inflammatory change with fibrosis. There were several areas of hyalinized collagenous plaques in keeping with the strong history of asbestos exposure, but no malignant disease. Post-operative recovery was complicated by myocardial infarction, pulmonary oedema, small pulmonary emboli, frank haematuria and continued weight loss. He then developed marked dependent oedema despite supportive enteral feeding via a nasogastric tube. Serum albumin was 22 g/l, urea and creatinine were within the normal reference ranges and urinalysis quantified 1.3 g/24 hours of proteinuria. The potential diagnoses were reviewed in light of the changing findings and discussed with the histopathologist. The original sample was reanalysed with Congo red stain. This is not part of our routine histological staining of pleural biopsies. The areas of fibrosis showed the classic apple green birefringence of amyloid deposition when viewed under high-intensity cross polarised light (Figure 2). A diagnosis of systemic AL amyloidosis was made. He was reviewed by specialist colleagues at the National Amyloidosis Centre and commenced on oral dexamethasone and melphalan. He remained unwell with poor performance status and despite supportive therapy died 4 months after diagnosis.

## Discussion

Amyloidosis is a multisystem disease characterised by the deposition of abnormal insoluble protein in tissues including the pleural space [1] disrupting organ function. Classically amyloidosis is divided into two main groups, AL amyloid in which the abnormal protein is from immunoglobulin light chain fragments or AA amyloid in which the fibrils are derived from serum amyloid A produced as a consequence of chronic inflammatory processes. MGUS in addition to progressing on to multiple myeloma is a recognised potential cause for systemic AL amyloidosis [2].

Pleural effusions in systemic amyloidosis are rare, large effusions, occupying over one-third of the hemithorax on chest radiograph were reported in 6% of cases of AL amyloidosis in the largest single centre experience published [3]. To be the presenting feature and source of the diagnostic biopsy for amyloidosis is unusual. Pleural effusions have often been considered purely epiphenomena secondary to cardiac dysfunction both systolic and diastolic, or proteinuria and hypoalbuminaemia due to renal involvement. The last review of amyloidosis and the respiratory tract in the journal of the British Thoracic Society, Thorax did not discuss the pleural space [4].

Direct amyloid infiltration of the pleural surfaces disrupting the balance between generation and removal of pleural fluid seems to be responsible for forming and maintaining pleural effusions in systemic amyloidosis. Cardiomyopathy plays a part in their development but is not sufficient in isolation [5].

In those with cardiac amyloid and ‘persisting pleural effusions’ (PPE), defined as being refractory to diuresis, Berk et al conclude that ‘neither echocardiographic measures of ventricular function nor the degree of nephrosis distinguished those with PPEs’. In addition those with PPEs ineligible for treatment had a very poor prognosis, with a median survival of just 1.8 months [3]. This is in keeping with our patient’s disease progression.

In conclusion, systemic amyloidosis should be considered as a potential cause of unexplained pleural effusions. It can be diagnosed on pleural biopsy, as emphasised by our experience it requires suspicion of the diagnosis in order the correct histological test is performed and it is difficult to treat. The case emphasises the importance of reviewing evolving diagnostic dilemmas in a multidisciplinary forum and reassessing old tests and samples in the light of a changing clinical picture.

## Abbreviations

AA amyloid: serum Amyloid A protein
AL amyloid: Amyloid Light chain
E/A ratio: Early/Atrial ratio
IgG: Immunoglobuin G
MGUS: Monoclonal Gammopathy of Undetermined Significance
PPE: Persisting Pleural Effusion

## References

[bib-001] Bontemps F, Tillie-Leblond I, Coppin MC (1995). Pleural amyloidosis: thoracoscopic aspects. Eur Respir J.

[bib-002] Sirohi B, Powles R (2006). Epidemiology and outcomes research for MGUS, myeloma and amyloidosis. Eur J Cancer.

[bib-003] Berk JL, Keane J, Seldin DC, Sanchorawala V, Koyama J, Dember LM, Falk RH (2003). Persistent Pleural Effusions in Primary Systemic Amyloidosis. Chest.

[bib-004] Gillmore JD, Hawkins PN (1999). Amyloidosis and the respiratory tract. Thorax.

[bib-005] Berk JL (2005). Pleural effusions in systemic amyloidosis. Curr Opin Pulm Med.

